# Self-Deception Reduces Cognitive Load: The Role of Involuntary Conscious Memory Impairment

**DOI:** 10.3389/fpsyg.2019.01718

**Published:** 2019-07-30

**Authors:** Zengdan Jian, Wenjie Zhang, Ling Tian, Wei Fan, Yiping Zhong

**Affiliations:** ^1^Cognition and Human Behavior Key Laboratory of Hunan Province, Hunan Normal University, Changsha, China; ^2^Department of Psychology, School of Education Science, Hunan Normal University, Changsha, China

**Keywords:** self-deception, deception, cognitive load, involuntary conscious memory, forward-looking paradigm

## Abstract

People often hear classic allusions such as plugging one’s ears while stealing a bell, drawing cakes to satisfy one’s hunger, and the emperor’s new clothes. These allusions reflect a principle that people believe in nonexistent phenomena to satisfy their desires, also called self-deception. The current research used three experiments to examine the impact of social status and cognitive load on self-deception, and further to explore the inner connection about cognitive load and self-deception. The results found that deceiving individuals of high social status can play a role through the intrinsic mechanism of involuntary conscious memory (ICM). The higher the cognitive load of the deceiver, the greater the possibility of deception. The study demonstrated that involuntary conscious memory is the internal mechanism of self-deception, further explore the origin of self-deception, and enrich the self-deception theory.

## Introduction

People often hear classic allusions such as plugging one’s ears while stealing a bell, pointing to a deer and calling it a horse, drawing cakes to satisfy one’s hunger, and the emperor’s new clothes. These allusions reflect the principle that people believe in nonexistent phenomena to satisfy their desires. This is called “self-deception.” Self-deception is a personality trait and an independent mental state, it involves a combination of a conscious motivational false belief and a contradictory unconscious real belief (von Hippel and Trivers, 2011). The forward-looking paradigm is widely used in self-deception research field to examine how self-deception influences predictions of the future (Chance et al., 2011; Yang, 2017; Ren et al., 2018; Liu et al., 2019). Participants take tests that assess their general knowledge and IQ. The paradigm includes three phases. In the first phase, participants are given the opportunity to view an answer key while taking an initial test; in the second phase, participants are asked to predict their future performance on a similar second test that lacks an answer key; in the third phase, participants take the second test, and the actual test score is recorded (Chance et al., 2011). Chance’s study found that compared with the control group, the self-deception group predicted significantly higher scores on the second test that were much higher than their actual test scores (the self-deception group had the opportunity to view an answer key while taking the initial test, whereas those in the control group did not have this opportunity). And a new study that used the forward-looking paradigm found that this paradigm can effectively indicate the existence of deception and that individuals of high social status are better able to control themselves and reduce self-deception (Ren et al., 2018).

With regard to the theoretical difference between deception and self-deception, previous studies have provided some explanations. Lu (2012) suggested that self-deception functions as a strategy in interpersonal communication to deceive others from the perspective of evolutionary theory. Because it is possible to deceive others directly, individuals can deceive themselves and then “honestly” send an incorrect message to the other party, such as withholding fitness-enhancing information from both oneself and others. Self-deception as an adaptation must cease to operate in most instances once the goal of deception has been achieved. Truthful information that has been kept from both oneself and others will then be retrieved to benefit the self. It is likely that this information manipulation co-opts memory to execute self-deception.

In human deception, cognitive load is an important indicator in recognizing deception (Trivers, 2011). Previous studies have shown that cognitive load reveals deception, but there are additional costs: the requirements of working memory reduce performance in challenging areas (Schmader and Johns, 2003) and damage social function (Hippel and Gonsalkorale, 2010). According to the theory of limited cognitive resources (Sweller, 1988), individual cognitive resources are generally limited. If too many cognitive resources are consumed, the cognitive load will be larger (Barrett et al., 2007; Van Dillen and Koole, 2007). While most people need to distinguish fact and lie, the deceiver needs to make sure that fact can be hidden and that lie can be supported. Two conflicting messages must exist at the same time, so a high cognitive burden is required. Based on the related research on self-deception and cognitive load, the current study proposed that, while self-deception provides a way to avoid this cognitive load, deceivers can convince themselves that their deception is indeed true, and they no longer need to maintain the truth of the event while highlighting the lie. On the contrary, by believing the lies they tell others, they can relax and focus on other things. Based on the theory of limited cognitive resources, can people reduce their cognitive load by deceiving themselves to avoid the cognitive cost of deceiving? This is the first question to be explored in our study.

Self-deception to some extent involves interpersonal self-deception. This process is achieved by relegating real information to the unconscious while consciously providing false information to others and to self (Trivers, 2000; von Hippel and Trivers, 2011). Some studies have found that when individuals use self-deception strategies to lie in interpersonal relationships, their situational pressures have considerable bearing on whether they use self-deception strategies. When the situation is more stressful, individuals are more likely to deceive themselves. Furthermore, high and low status relate to the level of one’s ability to detect lies (Lu and Chang, 2014; Ren et al., 2018). Previous studies on social status have shown that because low-status individuals lack power, if they wish to gain additional resources, they can only do so in a surreptitious way, such as hiding food, distracting others’ attention, or covering up their transgressions (Bugnyar and Kotrschal, 2004; Bräuer et al., 2007).

Previous studies have found that self-deception is related to memory. Roediger (1990) proposed that the memory structure consists of explicit/conscious and implicit/unconscious memory. Trivers (2000) proposed an information placement system of self-deception that supported Schacter and Roediger’s arguments. Conscious memory involves subjective awareness in the recollection of experience, whereas unconscious memory involves retrieval without awareness, which affects behavior. In interpersonal self-deception (Trivers, 2000), false information is in the conscious, whereas true information is in the unconscious. When the motivation for deception ceases, true information can return to the conscious.

Lu and Chang (2014) used Trivers’ theory to conduct empirical research using voluntary conscious memory (VCM) and involuntary conscious memory (ICM) to explore the relationship between social status and self-deception. VCM involves intentional and effortful recollections of experiences. By contrast, ICM involves unintentional and spontaneous recollections that are self-reported without effortful recall (Baddeley and Della Sala, 1996; Tulving, 2002). In widely adopted tasks of conscious memory, such as free recall and recognition, both VCM and ICM are assumed to be involved (Mandler, 1980; Schacter et al., 1989; Kvavilashvili and Mandler, 2004). An increasing number of studies of ICM have shown that spontaneous recollections in self-reports occur in various contexts, including semantic (Richardson-Klavehn and Gardiner, 1996; Kvavilashvili and Mandler, 2004) and episodic memory tasks (Berntsen and Jacobsen, 2008; Rasmussen et al., 2014). VCM and ICM may help to explain how conscious memory is temporarily impaired in self-deception. Self-deceivers make an effort to subjectively and voluntarily collect true information to convey to the deceived because self-deceivers are honest both to themselves and to the deceived. However, they unconsciously and involuntarily withhold true information from the deceived. Thus, the VCM of self-deceivers should be similar to that of nondeceivers, whereas ICM, which automatically emerges in the conscious without effortful recall, may be reduced to help achieve self-deception.

Based on the above research, we hypothesized that VCM and ICM may help to explain how conscious memory is temporarily impaired in self-deception. Lu and Chang (2014) found that during a task, in individuals with high social status, VCM will produce more memory error messages, while ICM can correct memory; that is, the high social status of individual participants is involved in self-deception. Therefore, the role of ICM between social status and self-deception remains to be verified.

Social status and cognitive load presumably have direct and indirect impacts on self-deception. What are the reasons and mechanisms that affect self-deception? This is the second question that this study aims to explore. Previous research showed that the impairment of ICM can reduce cognitive load, and self-deception can reduce cognitive load (Lu and Chang, 2014). The current study continues to explore this question: can this intrinsic mechanism reduce self-deception? Apart from the intrinsic mechanism, individual self-deception also affects cognitive load (Lu and Chang, 2014), which is an external factor that influences ICM. This study also aims to answer these questions. Based on Lu and Chang’s (2014) research, the purpose of the present study was to provide a more empirical test of Trivers’ theory by addressing the aforementioned issues.

From the above, the current study proposed three questions to explore the relationship between self-deception and cognitive load. First, people use self-deception to deceive others, whether self-deception can reduce cognitive load compared to direct deception? Second, if the results of Experiment 1 suggested that self-deception reduces an individual’s cognitive load, what is the inner mechanism? Previous studies have shown that interpersonal self-deception is that people will put real information into unconscious, while consciously providing false information to others and self (Trivers, 2000; von Hippel and Trivers, 2011), and previous study has shown that ICM can reduce the cognitive load (Lu and Chang, 2014). So, can memory impairment can achieve interpersonal self-deception? Third, according to the theory of limited cognitive resources, do individuals with high cognitive load experience self-deception due to excessive cognitive load? In other words, does cognitive load of an individual have an effect on the individual’s self-deception? In order to resolve the three questions, we designed three experiments.

## Experiment 1

In Experiment 1, the forward-looking paradigm was used to induce self-deception and deception to determine whether self-deception can eliminate the costly cognitive load associated with deception. We hypothesized that participants in the self-deception group and the deception group would have higher cognitive load scores compared to the control group. When comparing the two groups with high cognitive load scores, the self-deception group would have lower cognitive load scores than the deception group.

### Methods

#### Participants

The experimental procedure was approved by the IRB of the Institute of Psychology, Hunan Normal University. Ninety non-psychology-major students participated in the experiment. All participants provided verbal informed consent. Prior to this experiment, they had not taken civil service examinations or similar tests, and they signed informed consent for the experiment. Participants were randomly assigned to the self-deception group, the deception group, or the control group, with 30 people in each group (seven participants failed to understand the task’s rules and did not complete the task, so 83 participants with valid data were selected). We used G*Power Version 3.1.9.2 software (Faul et al., 2009) to acquire a *post hoc* calculation of the power of the sample size. According to the effect size of Experiment 1 (effect size *f* = 0.717), using the parameters *α* = 0.05, total sample size = 83, number of groups = 3, the analysis estimated a power of 0.99.

#### Measures for General Knowledge and Cognitive Load

The study used the forward-looking paradigm (Chance et al., 2011) and 20 general knowledge questions (Yang, 2017) for the experiment. An example of a general knowledge question was “When did the first world war break out? A.1910, B.1914, C.1939, D.1940.”

This experiment used the NASA-Task Load Index (NASA-TLX) scale developed by NASA, as translated and revised by Xiao et al. (2005), to measure the cognitive load after completion of the task. The NASA-TLX is a multidimensional instrument that consists of six subscales: Mental Demand (MD), Physical Demand (PD), Temporal Demand (TD), Frustration (FR), Effort (EF), and Performance (PE). Twenty-step bipolar scales are used to obtain ratings on these dimensions, resulting in a score between 0 and 100. The underlying assumption of the instrument is that the combination of these six dimensions is likely to represent the “workload” experienced by operators (Hart, 2006; Hoonakker et al., 2011).

#### Procedure

In this study, a single factor (self-deception, deception, and control group) was used in the design. The dependent variable was the cognitive load after the task was completed.

Prior to the experiment, the participants were told that the task was a general knowledge question in which a higher score indicated a higher level of intelligence. They were told that if their scores were in the top 20%, they would receive a bonus.

After the experiment began, the self-deception group completed five moderately difficult common-sense questions within 5 min. Each participant could see the answers, which were at the bottom of the test. After the first test, the participants were asked to complete 15 similar unanswerable common-sense questions. Prior to the second test, the participants were asked to make a prediction score and report it to the researcher. The participants then completed the second test.

The deception group was also able to see the answers at the bottom of the test. However, unlike the self-deception group, after completing all the tests, the participants checked their own answers and collated the scores of the second test. The researcher stressed that their answers would not be checked.

The control group could not see the answers. The control group also took the prediction test before the formal test, and the participants were required to make score predictions and report them to the researcher before completing the second test.

**Figure 1:**
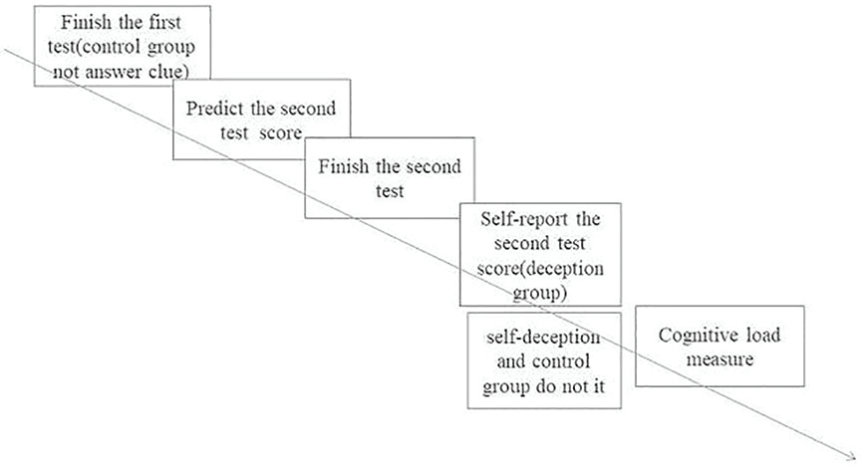
Trials of Experiment 1.

When the self-deception, deception, and control groups had completed the two tests, all participants were measured for the degree of cognitive load using the NASA scale. Because these tests did not involve physical exercise or performance in the experiment, these two sections of the NASA scale were eliminated (Figure 1).

### Results

#### Manipulation Check

There were significant differences in the prediction of the scores between the self-deception group and the control group on the second test, *F*(2,81) = 33.22, *p* < 0.001, $\eta_{p}^{2}$ = 0.45. Compared with the control group (*M* = 74.64, SD = 15.51) and the deception group (*M* = 84.04, SD = 10.20), the self-deception group (*M* = 102.76, SD = 13.34) reported higher predicted scores.

#### Cognitive Load Results

**Figure 2:**
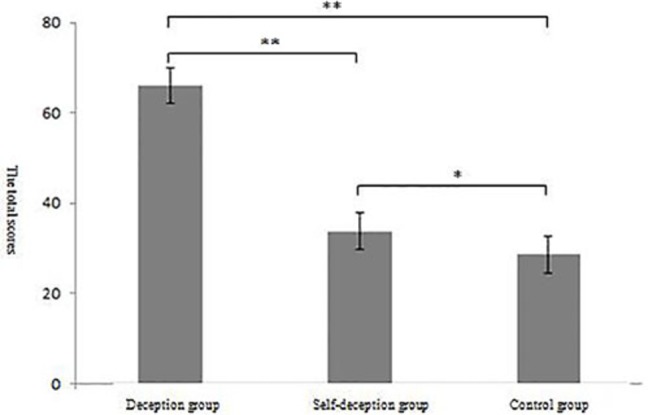
Results of Experiment 1: total scores of the deception, self-deception, and control groups (^*^p < 0.05, ^**^p < 0.01).

Next, the results of the NASA cognitive load scale of the self-deception group, the deception group, and the control group were calculated. The weight of the four questions in the NASA scale was 25% each, and the total score of the cognitive load was the sum of the weighted scores of each question multiplied by 10. The total scores of the cognitive load in the self-deception, deception, and control groups were analyzed by single-factor analysis of variance. The results showed that there were significant differences in the total score of the cognitive load among the three groups, *F*(2,80) = 11.29, *p* < 0.01, $\eta_{p}^{2}$ = 0.12. Multiple comparisons showed that the total cognitive load score (*M* = 62.02, SD = 9.51) of the deception group was significantly higher than that of the self-deception group (*M* = 33.79, SD = 9.22; *p* < 0.01) and of the control group (*M* = 28.571, SD = 8.99; *p* < 0.01). In addition, the self-deception group’s score was significantly higher than that of the control group (*p* < 0.05) (Figure 2).

### Discussion

The results of Experiment 1 showed that the predicted results for the second test in the self-deception group were significantly higher than the predicted results for the second test in the control group, indicating that self-deceptive behavior of the participants was successfully induced under the forward-looking paradigm. This result is consistent with previous conclusions (Yang, 2017; Ren et al., 2018) and indicates that both the forward-looking paradigm and the experimental materials could induce self-deception in the participants. In terms of the cognitive load results, the self-deception group experienced a greater cognitive load than the control group, indicating that the self-deception behavior itself, like all other ordinary behaviors, caused the participants to experience a certain cognitive load. However, compared with the self-deception group, the deception group had a greater cognitive load. This result is also in line with previous studies to some extent (Vrij and Barton, 2004; Atoum, 2006): under the same conditions of high cognitive load, the cognitive load of self-deception behavior is lower than that of deception. As evidence of the existence of interpersonal deception, self-deception has the advantage of saving cognitive resources and reducing cognitive load, as demonstrated by Experiment 1.

Experiment 1 showed that compared with the non-deception group, the deception and self-deception groups both experienced cognitive loads, but the cognitive load of the self-deception group was lower than that of the deception group. Previous studies have shown that when people of high social status are deceived, the memory adaptation of individual self-deception results in the impairment of ICM. We hypothesized that the memory of ICM is weakened by the self-adaptation of memory, thereby reducing the cognitive load of participants in the process of deception.

## Experiment 2

In Experiment 2, we hypothesized that compared with participants who deceived a low-status person, those who deceived a high-status person would temporarily be impaired in self-deception. Specifically, ICM would temporarily be impaired, but VCM would not be significantly different because the participants would consciously impair their memory to lie to others. We further hypothesized that both VCM and ICM would not be significantly different in the non-deception condition because the participants would not consciously impair their memory to lie to others.

### Method

#### Participants

The experimental procedure was approved by the IRB of the Institute of Psychology, Hunan Normal University. All participants provided verbal informed consent. One hundred non-psychology-major college students were recruited to participate in the experiment (*M*_age_ = 19.8 ± 0.75 years). They were paid 20 Yuan (approximately US$3) after the experiment, which lasted approximately 25 min. Participants were randomly assigned to four conditions: deception high status, deception low status, non-deception high status, and non-deception low status. We used G*Power Version 3.1.9.2 software (Faul et al., 2009) to acquire an *a priori* estimate of the required sample size. Using the parameters power = 0.8, effect size *f* = 0.25, *α* = 0.05 and given the current experimental design, the analysis estimated a sample size of 82. We ultimately recruited a total of 100 undergraduates. The actual power for this sample size was 0.88.

#### Experimental Material

The word materials were selected from 60 double-character Chinese words in the Chinese word library and edited by e-prime software. Each word was displayed on a computer screen for 7 s, followed by an instruction indicating whether the participant should cheat. The instructions for deception and non-deception were presented randomly, with half of the words being deceptive (including a deception instruction) and half being non-deceptive (not including a deception instruction). Participants who were assigned to deceive high-status individuals were told that they were going to deceive the teacher and were told to hide the words that had the “deceive the teacher” instruction in a later task. In contrast, the participants who were assigned to deceive low-status individuals were told that they would deceive students in later cheating tasks, which involved concealing words with the “cheat the student” instruction. Non-deceptive words had the instruction “not cheating” for the conditions of both high and low status.

The VCM and ICM measurements were based on the measurement method used in the research of Lu and Chang (2014). The cognitive load scale was the same as in Experiment 1.

#### Procedure

Experiment 2 used a 2 (Social status: High vs. Low) × 2 (Attribute of words: Deception vs. Non-deception) between-subjects design. The dependent variables were the number of test words of the first VCM/ICM test, the number of words in the second VCM/ICM test, and the difference in the number of words in the two VCM/ICM tests.

Before the experiment began, the participants were informed that they had been asked to participate in a deception task. Next, the participants were asked to remember 60 Chinese-language words (word memory tables) with medium frequency of use. They were told that the researcher would later ask them which words they had learned.

In the word memory table, half of the words included deception instructions, while the other half of the words did not. The deceptive words required participants to conceal the target words in the subsequent deception task, while the non-deceptive words required them to report the words honestly in the subsequent deception task. Participants who deceived high-social status individuals were told that their task was to deceive teachers in subsequent deception tasks. Those who deceived low-social status individuals were told that their task was to deceive students in subsequent deception tasks. At the end of the learning stage, to avoid rehearsal and recency effects, the participants completed a shape recognition filler task on the computer for 5 min.

After the filler task, the participants were told that they needed to complete a test before participating in the deception task. The VCM group completed the first VCM material test. The test content was a test paper containing 30 Chinese characters, with two spaces next to each of them. These Chinese characters were the first characters of the two-character words that the participants studied in the word memory table during the learning stage. The participants were asked to use the first character as a reminder to recall the words they had learned in the glossary and write them in the first space; using the first character to remember the second one is a “cued recall task.” If they could not remember the word they had learned, in the second space they were asked to write a word based on the two characters associated with the given first character. If the participant was able to actively and explicitly recall the word in the first space, the word that was written in the first space was considered to be recalled using VCM.

The ICM group completed the first ICM material test with the same test content as the VCM group. Unlike the VCM group, in the first space, the participants were asked to write the two-character words as quickly as possible. The participants were asked to do this quickly to avoid intentional recall. After completing the task, the participants were asked to check whether they had learned these words during the learning stage. If they confirmed learning a word, they were asked to write another two-character word beginning with the given character in the second space. The words written in the first blank and later identified as learning words were considered to be recalled using ICM because these words automatically reached the participant’s mind without deliberate recall. All participants completed the test and the cognitive load self-report scale.

**Figure 3:**
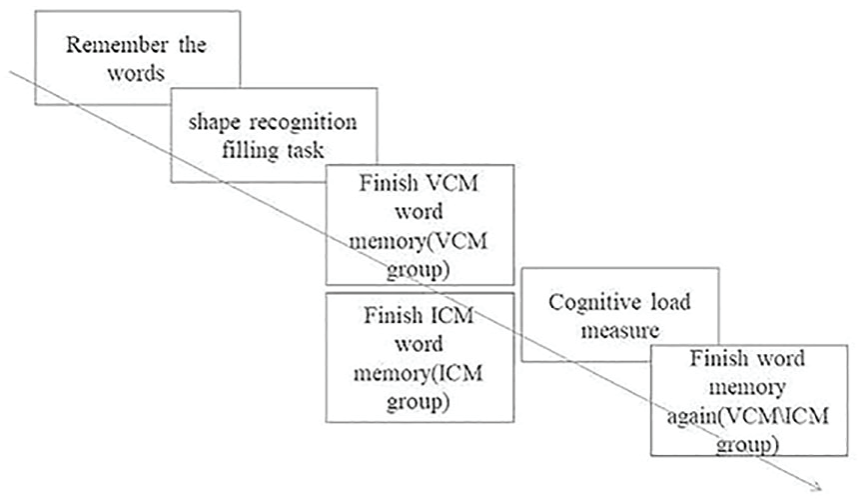
Trial of Experiment 2.

To test the participants’ actual memory of words, the participants were told that they would not have to cheat on the next task and should actually report the words they remembered. Finally, the participants were asked to take the VCM and ICM tests again. The content of the test was the remaining 30 words, excluding the words in the first test. At this time, the subjects were no longer in a situation of deception and self-deception and did not have a motivation to cheat (Figure 3).

### Results

#### Cognitive Load Check

A *t* test was conducted on the self-reported cognitive load scores of the high-social status and low-social status groups. The cognitive load scores of the high-social status group (*M* = 33.80, SD = 10.317) were significantly lower than those of the low-social status group (*M* = 57.54, SD = 13.459), *t*(98) = −9.899, *p* < 0.01, *d* = 0.50. These results suggest that people who cheat on high social status have a greater cognitive load.

#### Number of Words in the First Voluntary Conscious Memory/Involuntary Conscious Memory Test

A two-factor analysis of variance for VCM found that the main effect of social status and the attributes of words showed no significant difference.

**Figure 4:**
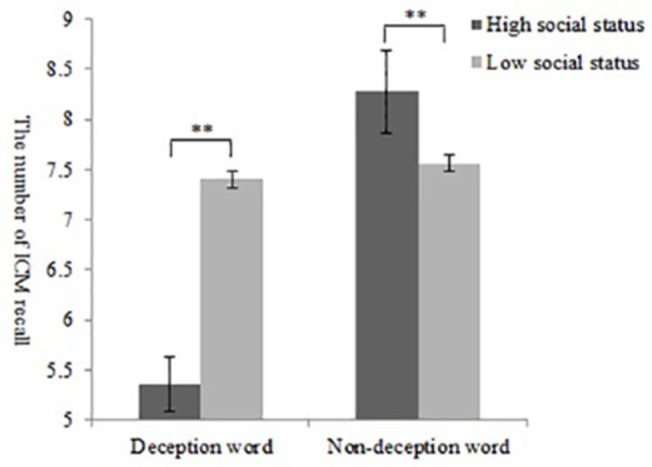
Results of Experiment 2: number of recall words in the first ICM test between the high- and low-social status group and the deception or non-deception word group (^**^ *p* < 0.01).

A two-factor analysis of variance for ICM found that the main effect of the attributes of words was significant, *F*(1,49) = 12.57, *p* < 0.01, $\eta_{p}^{2}$ = 0.17, and the number of ICM deceptive words was significantly lower than the number of ICM non-deceptive words. More importantly, there was an interaction between social status and the attributes of words, *F*(1,49) = 11.28, *p* < 0.01, $\eta_{p}^{2}$ = 0.11. Furthermore, a simple effect analysis showed that for the number of deceptive words, individuals who deceived people of high social status (*M* = 5.36, SD = 1.32) had significantly lower ICM recall than those who deceived people of low social status (*M* = 7.40, SD *=* 2.35) (Figure 4).

#### Difference in the Number of Words in the Second Voluntary Conscious Memory/Involuntary Conscious Memory Test

A two-factor analysis of variance for VCM found that the main effect of social status and the attributes of words showed no significant difference.

**Figure 5:**
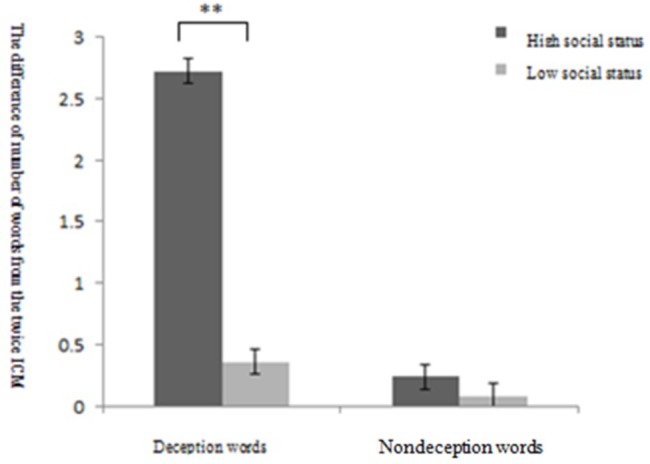
Results of Experiment 2: difference in the numbers of words between the two ICM tests between the high- and low-social status groups and the deception or non-deception words groups (^**^ *p* < 0.01).

A two-factor analysis of variance for ICM found that the main effect of word attributes was significant, *F*(1,49) = 13.85, *p* < 0.01, $\eta_{p}^{2}$ = 0.12. ICM deception word memory test (*M* = 3.08, SD = 0.33) was significantly higher than that of the non-deception test (*M* = 0.5, SD = 0.15); and the main effect of social status was significant, *F*(1,49) = 37.41, *p* < 0.01, $\eta_{p}^{2}$ = 0.28. Compared with the low social status deception (*M* = 0.41, SD = 0.13), the high social status deception had more ICM recall (*M* = 2.98, SD = 0.29) (Figure 5). More importantly, there was an interaction between the attributes of words and social status, *F*(1,49) = 21.68, *p* < 0.01, $\eta_{p}^{2}$ = 0.19. Comparing with non-deception words, Individuals who had cheated on high social status had more ICM recall (*M* = 2.67, SD = 0.23) than those who had cheated on low social status (*M* = 0.40, SD = 0.16) (Figure 5).

### Discussion

Experiment 2 found that in the first memory test, the number of deceptive words recalled by participants who deceived people of high social status was significantly lower than the number of words recalled by participants who deceived people of low social status. For the number of non-deceptive words, there was no significant difference between participants who deceived high- and low-social status individuals. The result is consistent with previous study, which showed that if individuals were confronted with deception targets who whose social status was higher than their own. High social status can induce self-deception, because of high social status represent authority and status, it was easier to evade the punishment of high-status individuals by using self-deception (Lu, 2012), it was easier to evade the punishment of high social status by using self-deception. Thus, individuals who were prone to more self-deception were of lower social status (Cummins, 1999; Lu and Chang, 2014). These results suggested that people used a self-deception strategy that is the impairment of ICM to achieve self-deception, and to better deceive others. Participants who deceived high-social status individuals with ICM in the task had more memory error messages than those with VCM in the task, who were able to correct their memory. This finding suggests that participants who deceived high-social status individuals engaged in self-deception.

In previous studies (Chance et al., 2011; Lu and Chang, 2014), a forward-looking paradigm was used to compare the ICM and VCM of self-deceiving individuals and deceiving individuals. Only self-deception ICMs were depleted. Thus, self-deception reduces the generation of cognitive load through the adaptiveness of memory compared to deceptive behavior. According to the theory of limited cognitive resources, there were limited effects accompanied by cognitive load consumption. Thus, individuals with a high cognitive load engage in self-deception because of the high cognitive load to alleviate this cognitive burden. To address this issue, Experiment 3 was designed to investigate how cognitive load affects an individual’s self-deception when an answer is provided.

## Experiment 3

Experiment 1 and Experiment 2 suggested that self-deception reduces cognitive load, which is caused by ICM impairment of self-deception. Because that we know that self-deception reduce cognitive load through ICM this intrinsic mechanism, what effect, in turn, does the level of an cognitive load have on self-deception? Therefore, Experiment 3 aimed to examine the effect of cognitive load on self-deception. We hypothesized that the difference between the second predicted score and the actual score of high cognitive load was greater than low cognitive load.

### Method

#### Participants

The experimental procedure was approved by the IRB of the Institute of Psychology, Hunan Normal University. All participants provided verbal informed consent. A total of 120 non-psychology-major college students were recruited to participate in the experiment (*M*_age_ = 19.68 ± 0.72 years). Prior to this, they had not participated in civil service examinations or similar tests, and they signed informed consent for the experiment. They were paid 20 Yuan (approximately US$3) after the experiment. We used G*Power Version 3.1.9.2 software (Faul et al., 2009) to acquire an *a priori* estimate of the required sample size. Using the parameters power = 0.8, effect size *f* = 0.25, *α* = 0.05 and given the current experimental design, the analysis estimated a sample size of 82. We ultimately recruited a total of 120 undergraduates, and the actual power for this sample size was 0.93.

#### Cognitive Load Task

The cognitive load task adopted the cognitive load scale (Greene et al., 2008). Based on the results of the preliminary experiment, we asked participants to memorize 12 numbers to distinguish between high and low cognitive load. The high cognitive load group memorized 12 different numbers, and the low cognitive load group memorized the same 12 numbers. The common-sense judgment material was the same as in Experiment 1.

#### Procedure

Experiment 3 used a 2 (cognitive load: High vs. Low) × 2 (Answer clue: Yes vs. No) between-subjects design. The dependent variables were second predicted score and difference between predicted and actual scores.

Prior to the start of the experiment, all participants were randomized into a high cognitive load group and a low cognitive load group. Participants in the high cognitive load group were assigned high cognitive load tasks, and participants in the low cognitive load group were assigned low cognitive load group tasks.

**Figure 6:**
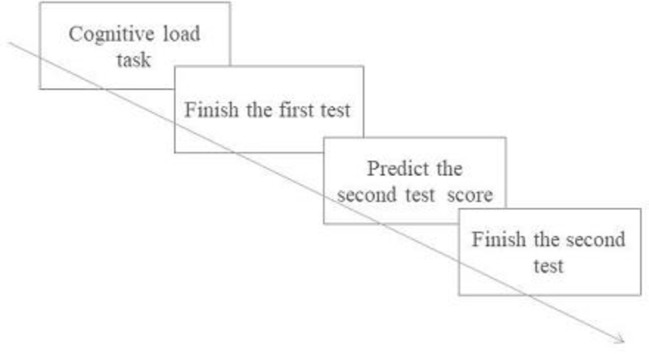
Trials of Experiment 3.

Next, the participants were asked to complete the initial five items of the general knowledge questions in 5 min. In the first test, participants were randomly divided into a group that received answer clues and a group that did not receive answer clues. Each group had 30 participants. The experimental group had the opportunity to see the answer clues on the bottom of the test paper on each page, but there were no answers at the bottom of the test for the control group. After completing the first test, all subjects answered a second test question without an answer prompt and then predicted the score of the second test (15 similar questions), wrote their predicted score on the test paper, and then finished the second test (Figure 6).

### Results

#### Second Predicted Score

The results showed that for the second predicted score, the main effect of the answer clues was significant, *F*(1,119) = 30.89, *p* < 0.01, $\eta_{p}^{2}$ = 0.24. The second predicted score was significantly higher for participants with answer clues (*M* = 107.25, SD = 17.09) than for those who did not have answer clues (*M* = 85.50, SD = 20.99). The main effect of cognitive load was significant, *F*(1,119) = 17.25, *p* < 0.01, $\eta_{p}^{2}$ = 0.29. The second test score for the high cognitive load group (*M* = 104.50, SD = 21.71) was significantly higher than that of the low cognitive load group (*M* = 88.25, SD = 19.20). The interaction between answer clues and cognitive load was not significant, *F*(1,117) = 0.10, *p* > 0.05, $\eta_{p}^{2}$ = 0.01.

#### Difference Between the Predicted and Actual Scores

**Figure 7:**
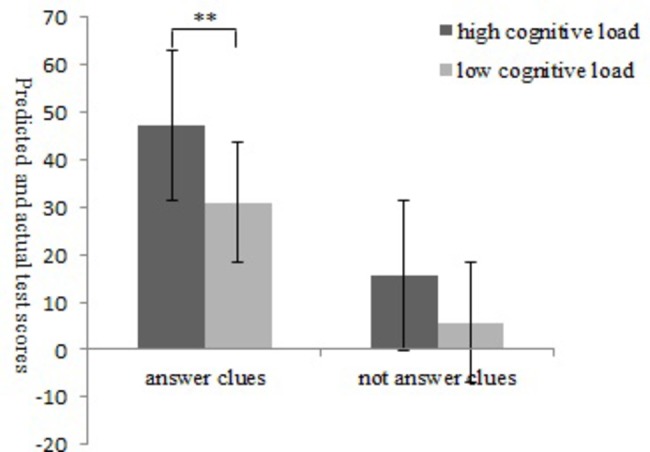
Results of Experiment 3: difference between predicted and actual scores for high versus low cognitive load and answer versus no answer clues (^**^ *p* < 0.01).

The results showed that for the second predicted score (the degree of self-deception), the main effect of the answer clues was significant, *F*(1,119) = 102.67, *p* < 0.01, $\eta_{p}^{2}$ = 0.47. Compared with the group that did not receive answer clues (*M* = 10.67, SD = 11.33), the answer clues group (*M* = 39.17, SD = 20.77) had significantly higher predicted scores than actual scores. The main effect of cognitive load was significant, *F*(1,119) = 18.03, *p* < 0.01, $\eta_{p}^{2}$ = 0.14. Compared with the low cognitive load group (*M* = 18.33, SD = 20.27), the high cognitive load group (*M* = 31.50, SD = 21.77) predicted scores were significantly higher than actual scores. The interaction between answer clues and cognitive load was significant, *F*(2,117) = 12.61, *p* < 0.01, $\eta_{p}^{2}$ = 0.11. Simple effect analysis showed that for the predicted and actual scores, comparing with non-answer clues, when answer clues, high cognitive load (*M* = 47.36, SD = 20.42) had significantly higher than low cognitive load (*M* = 30.98, SD = 2.35) (Figure 7).

### Discussion

Experiment 3 found that under the forward-looking paradigm of self-deception, individuals who received answer clues had significantly higher second test prediction scores than individuals who did not receive answer clues. Thus, the participants’ self-deception behavior was successfully induced under the forward-looking paradigm, a result consistent with previous research (Chance et al., 2011; Yang, 2017). Compared with the low cognitive load group, the second test prediction scores of the high cognitive load group were significantly higher, which showed that the high cognitive load group was more likely to experience an effect on cognitive load than the low cognitive load group. For the difference between the predicted score and the actual score on the second test, in the group with the answer clues, individuals with a high cognitive load had a greater difference in performance than those with a low cognitive load. Thus, individuals with a high cognitive load were more likely to engage in self-deception, which is consistent with our hypothesis.

The results of Experiment 3 also showed that individuals with a high cognitive load not only did not inhibit self-deception but also promoted self-deception. The reason may be that individuals with a high cognitive load were overburdened, and in cases where additional cognitive load consumption has been identified, they may have used the self-adaptability of memory and unconscious impairment to save cognitive resource consumption (Nairne and Pandeirada, 2010). ICM is the part of memory that is forgotten unconsciously. Forgetting part of the ICM produces less cognitive load. At the same time, people cannot recall the content of ICM; they can only lie to themselves, which promotes the behavior of self-deception. Therefore, cognitive load is not only an important indicator for distinguishing between self-deception and deceptive behavior but also an important external mechanism for self-deception behavior.

## General Discussion

### Self-Deception Saves Cognitive Resources More Than Deception

Experiment 1 showed that self-deception saves cognitive resources more than deception; it is consistent with previous research, the deceiver can be detected by clues associated with the cognitive load (Vrij and Barton, 2004; Atoum, 2006). Thus, self-deception is a strategy in interactions between individuals rather than an independent strategy. Because that interpersonal deception saving cognitive resources and reducing the production of cognitive load. In this study, the task of cognitive resource consumption was strictly controlled by direct laboratory operation. The intention was to induce self-deception and deception in groups under the same experimental conditions. Through subjective reporting of cognitive load, the cognitive load differences between the two behaviors were directly compared. This study found that under the same experimental conditions, the cognitive load generated by self-deception behavior was lower than that generated by deception behavior, which demonstrates that self-deception behavior, as a form of interpersonal deception, has the advantage of saving cognitive resources and reducing cognitive load.

### Memory Impairment May Be Internal Mechanism of Self-Deception to Save Cognitive Resources

Experiment 2 found that self-deception saved cognitive resources compared with deception, and it is necessary to discuss its internal mechanism. Experiment 2 found that the impairment of ICM deceived the individual memory system to save the internal mechanism of cognitive resources. Using the paradigm of the memory of self-deception behavior, the high or low social status of the target of deception induced different memory encoding processes in participants. This process can reveal the purposes of participants’ self-deception and deception behavior. Individuals who received a higher status target were exposed to higher potential lie detection rates and greater situational pressures, so they were more inclined to self-deception. In ICM measurements, fewer deceptive words were reported than non-deceptive words, demonstrating that self-deceiving individuals’ ICM was impaired. When instructed to deceive an individual of high social status, self-deceptive individuals also reported lower cognitive loads. These results were consistent with the hypothesis, showing that self-deception behavior as a means of interpersonal deception saves cognitive resources and reduces cognitive load. Thus, its internal mechanism seems to be the conscious memory impairment of the memory system. The impairment pattern of this memory component occurs during the individual’s encoding or retention of memory and is a silent and unheralded process of generation. This is what occurred in Experiment 2: individuals are often unaware of this deep change in memory, but this partial impairment of memory causes individuals to unconsciously “lie” to themselves because the memories that should have been kept are no longer there. This form of self-deception becomes a perfect pattern of deception.

### Cognitive Load Promotes Self-Deception

Experiment 3 showed that individuals with a high cognitive load did not inhibit self-deception but rather promoted it. This may be because individuals with a high cognitive load are overwhelmed by the burden of cognitive resource consumption. Research by Sweller (1988) suggests that the processing of information received by individuals produces a corresponding “level of mental energy” (Milton et al., 2008). ICM is a component of memory that is unconsciously impaired. When individuals lose part of their ICM, they have a lower cognitive load. At the same time, people cannot recall the content of nonrandom conscious memory; they can only lie to themselves, which promotes the formation of self-deception.

Experiment 3 addressed the problem of cognitive load as the external mechanism of self-deception behavior. Due to the limited amount of cognitive resources, individuals with a high cognitive load attempt to reduce it. One shortcut to achieve the goal of reducing cognitive load is to adapt to this conscious impairment. As a result, the scores predicted in the forward paradigm of self-deception were more daring and inaccurate and thus more prone to self-deception.

## Strengths and Limitations

The current studies explored the cognitive mechanism by which self-deception could reduce cognitive load and found that it was caused by impairing of involuntary conscious memory (ICM). The studies also found that self-deception would increase when individuals were under a high cognitive load. These results enrich previous research on self-deception field.

Even so, the current studies still have some limitations. First, because self-deception is very sensitive to the detection probability of presentation, it is difficult to verify self-deception through self-reports or other people’s observations (Lu and Chang, 2011). In our studies, it was inevitable to adopt a self-report’s method.

Second, in the process of Experiment 1, the experimenter found that when comparing the deception group with the self-deception and control group participants, there were larger mood swings and reactions in the deception group. Previous studies also found that emotion has a large influence on immoral behavior (Hess et al., 2018; Jahnke, 2018). Our study not involve other variables other than those discussed in the experiments. Future study could explore the influence of emotion.

Third, only the ways in which participants deceive themselves and hide information were studied. Deception and self-deception may also occur by means of distortion and forgery (Moomal and Henzi, 2000). Future research could study the multiple functions of deception.

## Conclusion

Self-deception reduces cognitive load. In terms of internal mechanisms, self-deceiving individuals use the self-adaptability of memory to achieve self-deception through the impairment of nonrandom conscious memory to reduce their cognitive load. In terms of external mechanisms, a higher cognitive load leads to more self-deception.

## Data Availability

The datasets for this manuscript are not publicly available because we propose to submit the dataset when the paper is accepted. Requests to access the datasets should be directed to fanwei@hunnu.edu.cn.

## Ethics Statement

This study was carried out in accordance with the recommendations of Hunan Normal University guidelines and written informed consent from all subjects. All subjects gave written informed consent in accordance with the Declaration of Helsinki. The protocol was approved by Hunan Normal University of the committee.

## Author Contributions

All authors were involved in all parts of the research. ZJ analyzed the data and wrote the manuscript. WZ designed studies and collected the data. LT performed the experimental procedures and examined the experimental material. WF and YZ guided experimental design and modified the manuscript.

### Conflict of Interest Statement

The authors declare that the research was conducted in the absence of any commercial or financial relationships that could be construed as a potential conflict of interest.
